# Delivery of a telehealth supported home exercise program with dietary advice to increase plant-based protein intake in people with non-alcoholic fatty liver disease: a 12-week randomised controlled feasibility trial

**DOI:** 10.1017/S0007114524000242

**Published:** 2024-05-28

**Authors:** Christine L. Freer, Elena S. George, Sze-Yen Tan, Gavin Abbott, Robin M. Daly

**Affiliations:** Institute for Physical Activity and Nutrition, Deakin University, Geelong, Australia

**Keywords:** Non-alcoholic fatty liver disease, Plant-based protein, Resistance exercise, Telehealth

## Abstract

This study evaluated the feasibility and safety of a telehealth delivered exercise plus plant-based protein diet in adults with non-alcoholic fatty liver disease (NAFLD). This was a 12-week, randomised controlled feasibility trial including twenty-eight adults aged > 45 years with NAFLD randomised to a home muscle strengthening program (3 d/week) with increased protein intake (target ∼1·2–1·5 g/kg/d) from predominately plant-based sources and behavioural change support (3–4 text messages/week) (Pro-Ex *n* 14) or usual care (UC, *n* 14). Feasibility was assessed via retention (≤ 10 % attrition), adherence (exercise ≥ 66 %; recommended daily protein serves ≥ 80 %) and safety (adverse events). Secondary outcomes included macronutrient intake (3 × 24-h records), weight, moderate-to-vigorous physical activity (MVPA) and 30 s sit-to-stand (STS) performance. Study retention was 89 %. Mean exercise adherence (Pro-Ex) was 52 % with one adverse event from 241 sessions. In Pro-Ex, mean daily plant protein serves increased (0·9 to 1·4/d) and animal protein decreased (1·5 to 1·2/d) after 12-weeks, but overall adherence (serves/day) was 32[RD1] % (plant) and 42 % (animal). Relative to UC, Pro-Ex experienced a mean 2·7 (95 % CI: 0·9, 4·4) increase in 30 s STS number, 46-minute (95 % CI: −153, 245) increase in MVPA, 1·7 kg (95 % CI: −3·5, 0·2) decrease in weight, 35·2 g (95 % CI: 11·0, 59·3) increase in protein. In adults with NAFLD a telehealth home exercise and dietary intervention was safe and improved habitual plant and animal protein intake, but overall adherence was modest suggesting more intensive healthcare support may be required.

Non-alcoholic fatty liver disease (NAFLD) presents as a serious global health issue estimated to affect approximately 30 % of adults worldwide^(1,2)^. This chronic liver disease is closely associated with unhealthy lifestyle behaviours including poor dietary habits, sedentary behaviours and physical inactivity^(3)^. Current clinical practice guidelines advise weight loss, primarily achieved through diet and exercise, for the management of NAFLD^(4)^, but adherence to such recommendations remains a challenge^(5)^. Telehealth has emerged as an encouraging approach to deliver and monitor personalised lifestyle programs that can significantly increase reach and accessibility for people with chronic diseases by overcoming common barriers including access to evidence-based programs, cost, time constraints and geographical location^(6–8)^. However, the limited digital health, lifestyle-based interventions conducted to date in those with NAFLD have reported mixed findings when examining the influence of text-message support, web-based platforms or apps designed to target weight loss, increasing physical activity or acceptability as an outcome^(9–12)^.

Although weight loss is at the forefront of NAFLD lifestyle recommendations, a common consequence of dietary-induced weight loss is a concurrent loss of muscle (lean) mass^(13)^, which has been shown to be a risk factor for both onset and progression of NAFLD^(14)^. Studies conducted in healthy adults and those with chronic conditions including NAFLD have shown that progressive resistance training (PRT) can increase muscle mass with some evidence that higher protein intakes can preserve or attenuate muscle loss during weight loss^(15–17)^. In healthy young and older adults, there is also evidence that the combination of PRT with a higher protein intake may provide additional benefits to muscle mass (and strength) compared with PRT alone, especially in those with low habitual intakes^(18)^. While most previous research has focused on animal protein and protein supplements such as whey protein, there is some evidence that plant-based sources of protein do not differentially impact PRT-related gains in muscle mass if the total dose of protein (∼1·6 g/kg/d) is comparable^(19,20)^.

For other health outcomes, there is growing evidence that the source of protein may be important^(21)^. For instance, foods of animal origin are a source of animal protein and saturated fatty acids which may increase insulin resistance and CVD risk while foods of plant origin are a source of plant proteins, fibre and bioactive compounds, which are associated with cardiometabolic benefits^(22,23)^. Data from population-based studies suggest a high consumption of animal protein (especially red and/or processed meat) may increase the risk of NAFLD^(24,25)^, while greater intake of plant proteins and fish may be protective^(26,27)^. A diet that emphasises plant sources of protein is also in line with the Mediterranean diet composition that is recommended in NAFLD clinical guidelines^(4)^. Therefore, it is likely that lifestyle strategies that encompass PRT combined with a higher total protein intake may be beneficial for optimising body composition, while a higher protein intake from predominately plant-based sources may provide other potential cardiometabolic health benefits in those with NAFLD. However, to our knowledge no study has assessed the feasibility and safety of a lifestyle intervention delivered and monitored by healthcare professionals via telehealth incorporating a home-based, muscle strengthening program combined with a higher protein intake from predominately plant-based sources in adults with NAFLD.

The aim of this randomised controlled trial was to evaluate the feasibility (retention and adherence) and safety of a 12-week telehealth, home-based exercise and nutritional support program incorporating a personalised muscle strengthening exercise program and healthy eating recommendations focusing on a higher protein intake from predominately plant-based sources in middle and older aged adults with NAFLD. Secondary exploratory aims were to evaluate changes in macronutrient intake including protein intake from different protein sources (plant and animal), anthropometric measures, habitual physical activity and physical function.

## Methods

### Study design

This was a 12-week randomised controlled study investigating the feasibility and safety of delivering an exercise, dietary and behavioural change support intervention via telehealth to middle-aged and older adults with NAFLD. Following baseline assessments, participants were randomised (in blocks of 2), stratified by age (< 65 years, ≥ 65 years), by an independent researcher not directly involved in the study, to receive either the telehealth exercise and protein (Pro-Ex) intervention or usual care (controls). Study recruitment occurred over an 8-month period (May 2022 to November 2022) with the intervention delivered in two cohorts: cohort 1 October 2022 to December 2022; cohort 2 November 2022 to February 2023. Research staff and participants were not blinded to the group allocation given the nature of the intervention to educate and adopt diet and exercise changes. Participants were assessed at baseline, 6 and 12 weeks, with exception of protein intake that was also assessed at weeks 2 and 9. All assessments and training were performed remotely at the participant’s home. The study was conducted according to the guidelines laid down in the declaration of Helsinki, and all procedures involving human subjects were approved by the Deakin University Human Research Ethics Committee (2021-359) and prospectively registered with the Australian and New Zealand Clinical Trial Registry https://www.anzctr.org.au (ACTRN12621001706864). Written informed consent was provided (electronically) by all participants prior to participation.

### Participants

**Fig. 1.**
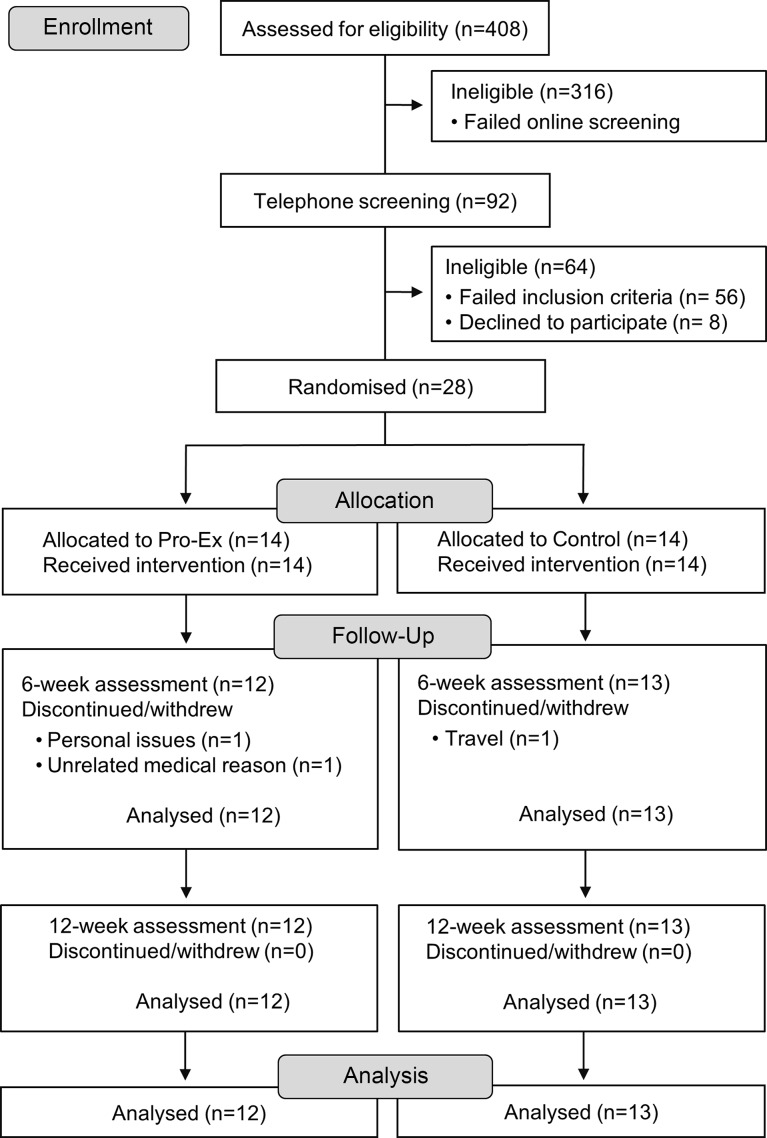
Study flow chart. Pro-Ex, protein plus exercise.

A total of twenty-eight men and women aged ≥ 45 years with self-reported NAFLD, as diagnosed by a doctor, determined via routine ultrasound or biopsy and/or at least one elevated serum alanine aminotransferase level (> 20 U/L female, > 30 U/L male), were recruited from social media (Facebook), general practice and liver clinics throughout Australia. We acknowledge the recent guidelines and associated change in nomenclature definition of metabolic dysfunction-associated steatotic liver disease (MASLD); however, registration of the study protocol (13/12/2021) and participant recruitment preceded consensus and publication of the new definition. Participants were excluded based on the following criteria: current/prior involvement in PRT (> 1/week) or moderate-intensity physical activity (> 150 min/week) in the last 3 months; self-reported weight loss/gain of > 5 kg in the last 3 months; renal disease or impairment, orthopaedic, cardiovascular or respiratory disease preventing participation in the intervention or those with absolute contraindications to exercise based on the American College of Sports Medicine guidelines^(28)^; no evidence of another form of liver disease; currently following a vegetarian or vegan diet; intake of ≥ 2 serves of protein rich (fortified) supplements per week; an average intake of ≥ 1 (female) or ≥ 2 (male) alcoholic drinks per day and currently undertaking a dietary intervention from a dietitian/nutritionist. Participants were also required to obtain consent from their consulting physician (if they answered yes to any of the contradictions to exercise)^(28)^, and they needed to possess a mobile phone, tablet or computer with text message functioning, home internet connection and own or have access to scales suitable for measuring body weight. All participants were initially screened via an online questionnaire (*n* 408), with participants meeting the initial eligibility criteria (*n* 92) undertaking further screening via telephone. A total of thirty-six participants were deemed eligible, with twenty-eight participants agreeing to participate in the study and who were randomised to either group (Fig. 1).

#### Exercise intervention

Participants allocated to the Pro-Ex group were prescribed a 12-week muscle strengthening program by a qualified accredited exercise physiologist (EP) using a commercially available web-based exercise prescription platform and participant app (TeleHab, VALD Health) accessible via their phone or tablet. The TeleHab app provided participants with narrated videos of all exercises to demonstrate correct technique, and participants were asked to rate their perceived exertion (RPE) and any pain (both on a scale of 0–10) upon completion of each exercise and provide any written comments regarding their prescribed exercises, all of which were monitored remotely by the EP thrice weekly to check on participants progress and promote safety. All exercise sessions were saved in the TeleHab platform to monitor adherence and progress. Exercise prescription was personalised by the EP based on each participant’s baseline assessment and medical and physical activity history. Participants were prescribed an ∼25-minute whole body muscle strengthening program consisting of 6–8 exercises utilising major muscle groups (two sets of ten repetitions for each exercise) at a moderate intensity (5 ‘hard’ to 8 ‘very hard’ RPE scale) to be completed thrice weekly (on non-consecutive days). In week 6, the EP prescribed (via TeleHab) a new program (set of exercises) for the remaining 6 weeks of the intervention. Some examples of the prescribed exercises include sit-to-stands, squats, lunges, wall/inclined push-ups, upright row (resistance band) and triceps extension (resistance band). Prior to commencing the programme, participants attended an online (Zoom) consultation with the EP to receive training on using the TeleHab app, tips for exercising safely at home and to ensure participants were performing the prescribed exercises correctly. In weeks 2 and 6 participants attended small group (∼2–3 participants) consultations (via Zoom) with the EP who was able to answer any questions and promote motivation and a sense of community for participants. In addition, all participants were instructed and encouraged to engage in aerobic activity (e.g. brisk walking) twice weekly (total ≥ 60 min per week) throughout the 12-week program, which they recorded in a daily activity calendar.

#### Dietary intervention

For the 12-week intervention, an isoenergetic healthy eating plan was devised by a nutritionist which emphasised a protein intake of 1·2–1·5 g/kg/d from predominately plant-based foods (legumes, tofu, nuts, peanuts and seeds), as well as fish and white meat sources. This was translated into recommendations of at least three serves/day of protein from all sources consisting of two serves from plant sources and 1–1 ½ serves from animal-based protein sources, which were based on each participant’s age and sex. Serving guidelines were then provided regarding daily protein from plant sources (total 2 serves/d from nuts and seeds and/or tofu/legumes and/or beans) and animal sources (total 7–10 ½ serves/week from recommendations for seafood (≥ 2 serves/week), poultry (≤ 2 serves/week) eggs (2–4 serves/week), red meat (< 2 serves/week) and processed meat (≤ 1 serve/week)). Protein intakes (plant and animal sources) were recorded via an online protein checklist at baseline to guide participant recommendations about their healthy eating plan. Further checklists were completed in weeks 2, 6, 9 and 12 to monitor adherence. Dietary recommendations were based on a heart healthy, modified Mediterranean style dietary pattern informed by current clinical guidelines for NAFLD^(4,29)^. In week 1, participants received written resources providing information on protein serving sizes, recommended servings from different protein sources, sample meal plans and plant-based recipes. Further education about the healthy eating plan and how to complete the 24-h record were also delivered in week 1 via an online (Zoom) consultation with the study nutritionist. At week 2 and 6, participants attended small group (∼2–3 participants) online (Zoom) consultations with the nutritionist to receive information and tips on increasing plant-based protein intake and adhering to protein serving recommendations, feedback on protein checklists and motivational tips to adopt the healthy eating plan.

#### Behavioural change support

In addition to receiving regular support from the EP and nutritionist, participants in the intervention group received regular text messages (3–4 messages/week) with health focused and motivational messages to support behavioural change. This included reminders and tips for increasing daily serves of protein (predominately plant-based) and adhering to other dietary recommendations, physical activity/movement and more generic messages providing motivational support.

#### Control group (usual care)

Participants assigned to the control group received usual care from their general practitioner or liver clinic specialist. They also received generic handouts at the start of the study providing information on the importance of healthy eating (increasing fruits (≥ 2 serves) and vegetables (≥ 5 serves), choosing wholegrains, lean proteins and reduced fat versions of dairy foods and healthy mono and polyunsaturated fats and limiting processed foods) based on recommendations from the Australian Heart Foundation^(30)^ and being physically active as per the Australian Physical Activity Guidelines^(31)^.

### Measurements

#### Feasibility

Feasibility was assessed via retention and adherence as outlined below.

##### Retention

Retention was calculated as the proportion of participants who completed the 12-week intervention. Acceptable retention for this study was defined as < 10 % attrition.

##### Adherence

Adherence was recorded for both the exercise and dietary recommendations as follows; (1) the total number of exercise sessions completed relative to the total number prescribed (expressed as a percentage) and (2) the number of serves of protein-rich foods consumed relative to the number recommended from plant-based protein sources (total 2 serves per day from nuts and seeds and/or tofu and legumes and/or beans) and animal protein sources (7–10 ½ serves per week from seafood and/or eggs and/or poultry and with less than 2 serves from red meat and less than or equal to 1 serve from processed meat) and total protein (those participants meeting both the above daily plant and weekly animal serving recommendations). Acceptable adherence for this study was defined as at least 66 % (two out of three sessions/week) to the muscle strengthening exercise program and ≥ 80 % adherence (to ensure participants protein intake reached at least 1·0–1·2 g/kg/d) to the recommended serves (average over 12-weeks) of both plant and animal protein sources assessed via protein checklists in weeks 2, 6, 9 and 12. For example, if the recommendation was ≥ 2 serves/d, adherence would require an average intake of at least 1·6 serves/d. For comparison, adherence to the above dietary recommendations was also reported for controls.

##### Adverse events (safety)

An adverse event was defined as any intervention-related event that results in withdrawal or modification to the exercise or healthy eating program. Participants in the Pro-Ex group were asked to record any adverse events directly into TeleHab so that they could be reviewed by the research investigators. Information on any potential adverse events were also collected by the EP and nutritionist during the video consults in weeks 2 and 6.

#### Additional measures

##### Dietary assessment

Changes in macro and micronutrient intakes and food groups were assessed via three separate 24-h food records completed at baseline, 6 and 12 weeks. Food records were completed using the commercially available web-based dietary app (Research Food Diary) accessible via the participant’s phone or tablet. Dietary information was analysed using Foodworks nutrient analysis software (Xyris).

##### Anthropometry

Due to the telehealth nature of this study, anthropometry measures (height, body weight and waist circumference) were self-reported at baseline, 6 and 12 weeks. Participants were sent tape measures and provided written instructions on how to measure their waist circumference (in cm). Body mass index (BMI) was calculated as weight (kg) divided by height (m^2^).

##### Physical activity

The validated short International Physical Activity Questionnaire was used to assess physical activity^(32)^. Questions covered the frequency, intensity and duration of walking, moderate and vigorous physical activity and total time spent sitting per day (sedentary behaviour) over the past 7 d. Total time (min/week) spent walking and in moderate-to-vigorous activity and sedentary time were calculated at baseline, 6 and 12 weeks.

##### Physical function

Physical function was assessed using the 30 s sit-to-stand (STS) test. Participants were asked to use a firm chair, approximately 46 cm high, with no wheels or arm rests, with the same chair used for each assessment. Participants were shown a demonstration video of the 30 s STS test, then instructed to place their chair against a wall and position their device (e.g. smart device or laptop) ∼2·0 m away from the chair at an angle of ∼45° and adjust their device so the researcher could see their entire body and chair. Participants started the test from a seated position with hands across their chest and on the command ‘go’ were instructed to stand fully upright, then return to a seated position repeatedly for 30 s, with the final score being the number of completed STS in 30 s counted by the researcher. The 30 s STS has been shown to be a valid indication of lower limb strength^(33)^ and is feasible and safe when delivered and evaluated via telehealth as a remote assessment of physical function^(34)^.

##### Medical and health history

History of chronic diseases (hypertension, CVD, lung disease, diabetes and kidney disease), smoking (never, previous and current smoker), current medications (number and type) and education (high school, trade/technical, university/tertiary) were evaluated by a lifestyle questionnaire competed at baseline.

### Statistical analysis

As this was a feasibility study, no formal sample size calculations were required as per current recommendations,^(35)^ and significance testing was not conducted. Descriptive variables were calculated using Stata/se software version 17.0 (StataCorp), and all data were reported as mean with sd, median with interquartile range or number with proportions, unless stated. For the exploratory outcomes of physical activity, 30 s STS performance and anthropometric measures and effect sizes (Cohen’s d) were calculated to explore trends for between-group differences for the changes over 12 weeks. Within-group changes were calculated as the mean change (absolute or percentage) from 6 and 12 weeks compared with baseline. Between-group differences for the change were calculated as the mean difference between groups for the change from 6 or 12 weeks compared with baseline. Effect sizes for between-group differences for the changes from baseline were calculated according to the mean change in Pro-Ex minus the mean change in controls divided by the baseline pooled sd of both groups. Cohen’s d values of < 0·2, 0·2 to < 0·5, 0·5 to < 0·8 and ≥ 0·8 were used to demonstrate trivial, small, moderate and large effect sizes, respectively^(36)^.

## Results

The baseline characteristics of participants are shown in Table 1, with groups being similar with the exception that participants in Pro-Ex had a higher weight and BMI than controls. The mean ± sd age of all participants was 63·5 ± 7·3 years (range 47–77 years) with 93 % being female and 36 % (*n* 10) classified as overweight (BMI 25–29·9 kg/m^2^) and 54 % (*n* 15) as obese (BMI ≥ 30 kg/m^2^). A total of twenty-two (79 %) participants reported taking a prescribed medication, with the mean number for those prescribed medication being 2·1 ± 1·9. Overall, 46 % (*n* 13) of participants reported the presence of one or more chronic disease, which included CVD (*n* 1), hypertension (*n* 10) and diabetes (*n* 2).

**Table 1.** Participant characteristics

	Pro-Ex			Control		
	Mean		sd	Mean		sd
*n*	14			14		
Females, *n*	12			14		
Age, years	63·2		7·7	63·9		7·2
Weight, kg	92·5		24·5	78·8		17·9
Height, cm	165·1		8·4	158·9		9·0
BMI (kg/m^2^)	33·7		7·7	31·1		5·7
Waist circumference, cm	110·4		16·3	102·1		14·1
	*n*	%		*n*	%	
Smoking status						
Never smoked	12	85·7		13	92·9	
Previous smoker	2	14·3		1	7·1	
Current smoker	0	0		0	0	
Highest level of education						
High school	3	21·4		2	14·3	
Trade/Technical	3	21·4		4	28·6	
University/Tertiary	8	57·1		8	57·1	
Presence of chronic disease(s)^*^	6	42·9		7	50	
Prescribed medication	12	85·7		10	71·4	
	Mean		sd	Mean		sd
Number of medications	2·1		1·8	2·1		2·0

Pro-Ex, protein plus exercise. All values are expressed as mean and sd or number (%).

* Presence of chronic diseases include self-reported hypertension, CVD and/or diabetes.

### Adverse events

Over the 12-week program, there was one minor exercise-related adverse event (musculoskeletal complaint of knee pain related to a pre-existing knee injury) reported from a total of 241 completed exercise sessions. The participant continued to exercise with a modified program for 2 weeks before returning to the full program.

### Feasibility

#### Retention

Study retention was 89 % (25 of 28 participants completed the study) which was similar for Pro-Ex (86 %) and Con (93 %). Three participants (Pro-Ex group *n* 2, control *n* 1) withdrew during the first 6 weeks due to personal issues, an unrelated medical reason and overseas travel (Fig. 1).

#### Attendance at consultations

All participants in Pro-Ex (*n* 14) attended the initial one-on-one sessions with their EP and study nutritionist at the start of the study. Attendance at the small group sessions in week 2 with both the EP and nutritionist was 86 % (*n* 12), while in week 6, attendance was 64 % (*n* 9) and 71 % (*n* 10), with the EP and nutritionist, respectively, for the eleven participants in Pro-Ex that remained in the study.

#### Exercise adherence

The mean ± sd adherence to the thrice weekly muscle strengthening program (Pro-Ex) was 52 ± 36 % (median 55 %, interquartile range 15–69 %). This included two participants who withdrew at weeks 3 and 5, three participants who stopped training within the first three weeks and two participants who completed more sessions than they were prescribed (108 % and 121 % adherence). A further two participants reported knee (*n* 1) and back (*n* 1) pain unrelated to the exercise program, which resulted in one participant stopping the exercise program in week 3, and the second participant exercising at a reduced capacity in weeks 9–11. Overall, 36 % (*n* 5) of participants completed, on average, < 1 session per week, 29 % (*n* 4) completed 1–< 2 sessions per week and 36 % (*n* 5) completed ≥ 2 sessions per week. When assessing only those participants who completed the 12-week exercise program (nine of fourteen participants), the mean ± sd adherence to the muscle strengthening program was 73 ± 26 % (median 64·7 %, interquartile range 62–74 %). The mean ± sd adherence to the twice weekly (at least 60 min in total) aerobic exercise recommendations was 52 ± 41 % (median 63 %, interquartile range 0–100 %).

#### Dietary adherence

Overall, the mean proportion of participants in the Pro-Ex group who met (at least 80 %) of the plant protein (serves/day) and animal protein (serves/week) recommendations over the 12-week intervention was 32 % and 42 %, respectively, with only 14 % (*n* 2) of participants meeting the total protein recommendations for both animal and plant sources (Table 2). Adherence to the plant protein serves/day was highest in week 6 (50 %), before decreasing slightly at weeks 9 and 12 (33·3 %), while adherence to the recommended serves/week of animal protein increased from 14·3 % at baseline to 57·1 % in week 2 and then varied between 16·7 and 58·3 % throughout the rest of the intervention. The proportion of control participants whose habitual plant protein intake met recommended serves/day throughout the study period was 0 % while the proportion who met the recommended serves/week for animal protein varied from 15·4 to 38·5 % throughout the 12-weeks. There were no control participants who met both the recommended plant and animal protein serve at any time.

**Table 2.** The number and proportion (%) of participants who adhered to at least 80 % of the recommended serves and the mean daily serves of plant, animal and total protein at baseline and throughout the 12-week intervention in the Pro-Ex and control group

	Plant protein				Animal protein				Total protein			
	Adherence		Serves/day		Adherence		Serves/day		Adherence		Serves/day	
	*n*	%	Mean	sd	*n*	%	Mean	sd	*n*	%	Mean	sd
Baseline												
Pro-Ex	2	14·3	0·9	1·1	2	14·3	1·5	0·7	0	0	2·4	1·7
Control	0	0	0·5	0·3	4	28·6	1·5	0·6	0	0	1·9	0·5
Week 2												
Pro-Ex	2	14·3	1·2	0·8	8	57·1	1·1	0·5	2	14·3	2·3	1·0
Control	0	0	0·5	0·3	2	15·4	1·4	0·4	0	0	1·9	0·4
Week 6												
Pro-Ex	6	50·0	1·6	0·8	4	33·3	1·6	0·6	2	16·7	3·2	1·1
Control	0	0	0·4	0·4	2	15·4	1·4	0·5	0	0	1·8	0·6
Week 9												
Pro-Ex	4	33·3	1·4	0·6	2	16·7	1·6	0·5	0	0	3·0	0·9
Control	0	0	0·5	0·4	5	38·5	1·3	0·6	0	0	1·9	0·9
Week 12												
Pro-Ex	4	33·3	1·4	0·7	7	58·3	1·2	0·4	3	25·0	2·6	0·8
Control	0	0	0·5	0·3	3	23·1	1·2	0·4	0	0	1·7	0·4
Mean												
Pro-Ex	4	32·0	1·4	0·7	5	42·0	1·4	0·5	2	14·0	2·8	1·0
Control	0	0	0·5	0·3	3	23·0	1·3	0·5	0	0	1·8	0·6

Pro-Ex, protein plus exercise group. All values are expressed as number and proportions (%) or mean and sd. The number (and proportion) of participants classified as adhering to the plant protein recommendations was based on meeting ≥ 80 % of the 2 serves/d. For adherence to the animal protein recommendations this was based on meeting ≥ 80 % of the 7–10 ½ serves/week which included from seafood and/or eggs and/or poultry and with less than 2 serves from red meat and less than or equal to 1 serve from processed meat per week. For total protein adherence this included meeting both plant and animal protein recommendations. Pro-Ex - baseline and week 2 (*n* 14) and weeks 6, 9 and 12 (*n* 12); Controls – baseline (*n* 14) and weeks 2, 6, 9 and 12 (*n* 13). Mean represents average from weeks 2 to 12.

#### Daily serves of total, animal and plant protein

Despite modest adherence to the specific protein (plant and animal) recommendations, participants in Pro-Ex did display some improvements in their daily serves of total and plant-based protein (Table 2). Overall, the mean ± sd serves/day of total protein for Pro-Ex increased from 2·4 ± 1·7 at baseline to 3·2 ± 1·1 at week 6 before decreasing to 2·6 ± 0·8 in week 12. In controls, mean serves/day of total protein remained relatively stable throughout the study (range 1·7 ± 0·4 to 1·9 ± 0·5 serves/d). Pro-Ex also increased their mean serves/day of plant protein from 0·9 ± 1·1 at baseline to 1·6 ± 0·8 at week 6 and 1·4 ± 0·7 at week 12, while intakes for controls remained stable (∼0·5 serves/d). Regarding serves/day of animal protein, mean intakes were comparable between Pro-Ex and controls at baseline (1·5 ± 0·7 *v*. 1·5 ± 0·6) and decreased slightly in both groups at week 12 (both 1·2 ± 0·4 serves/d).

#### Serves of protein from different plant and animal sources

The number of mean weekly serves of protein from different sources and proportion of participants meeting ≥ 80 % adherence to the recommended serves throughout the study for Pro-Ex and controls is shown in Table 3. Overall, throughout the intervention, Pro-Ex increased their intake of seafood (mean change 1·3 serves/week), nuts and seeds (0·2 serves/d) and legumes (0·3 serves/d) and reduced their intake of red meat (0·6 serves/week) and processed meat (1·2 serves/week). For controls, intakes from both plant (serves/day) and animal-based sources (serves/week) of protein remained relatively consistent over the 12-week intervention. In terms of the proportion of participants achieving ≥ 80 % adherence to our recommendations for specific protein sources, there was little or no change for participants in Pro-Ex with regards to nuts and seeds, eggs and poultry but a higher proportion of participants achieved the recommendations for legumes and tofu (7–32 %), seafood (50–78 %), red meat (71–86 %) and processed meat (36–66 %) (Table 3). For controls, there were no discernible changes in the proportion of participants meeting the recommendations.

**Table 3.** Mean daily plant-based and weekly animal-based serves of protein from different protein sources and the proportion of participants meeting ≥ 80 % adherence to the recommended intakes in Pro-Ex and controls

		Baseline		Week 2		Week 6		Week 9		Week 12		Mean	
		Mean	sd	Mean	sd	Mean	sd	Mean	sd	Mean	sd	Mean	sd
Nuts and seeds (≥ 1 serve/d)													
Pro-Ex	Serves/day	0·5	0·6	0·6	0·5	0·8	0·5	0·6	0·3	0·7	0·5	0·7	0·4
	% Adherence	28·6		28·6		41·7		25·0		25·0		30·0	
Control	Serves/day	0·3	0·2	0·3	0·3	0·3	0·2	0·4	0·3	0·3	0·2	0·3	0·3
	% Adherence	7·1		7·7		0		15·4		0		5·8	
Legumes and tofu (≥ 1 serve/d)													
Pro-Ex	Serves/day	0·4	0·6	0·7	0·5	0·8	0·5	0·7	0·5	0·6	0·4	0·7	0·5
	% Adherence	7·1		28·6		41·7		33·3		25·0		32·0	
Control	Serves/week	0·1	0·1	0·2	0·2	0·1	0·2	0·1	0·2	0·2	0·2	0·1	0·2
	% Adherence	0		0		0		0		0		0	
Seafood (≥ 2 serves/week)													
Pro-Ex	Serves/week	2·0	1·9	2·8	2·2	4·0	3·3	3·4	2·3	2·9	1·6	3·3	2·4
	% Adherence	50·0		78·6		75·0		75·0		83·3		78·0	
Control	Serves/week	2·1	1·8	2·7	1·8	2·1	1·4	2·4	1·6	1·9	1·5	2·3	1·5
	% Adherence	57·1		76·9		69·2		69·2		61·5		69·2	
Eggs (2–≤ 4 serves/week)													
Pro-Ex	Serves/week	1·9	1·0	1·5	0·9	2·4	1·6	2·4	1·7	1·8	1·2	2·0	1·4
	% Adherence	71·4		57·1		83·3		66·7		50·0		64·0	
Control	Serves/week	2·1	1·0	2·5	1·6	2·4	1·5	2·3	1·7	1·3	0·9	2·1	1·5
	% Adherence	78·6		76·9		84·6		53·8		46·2		65·4	
Poultry (≤ 2 serves/week)													
Pro-Ex	Serves/week	3·0	3·6	1·7	1·1	2·3	1·3	2·9	2·1	1·8	0·8	2·2	1·4
	% Adherence	64·3		78·6		66·7		58·3		83·3		72·0	
Control	Serves/week	2·5	1·9	2·3	1·0	2·6	1·2	2·3	1·4	2·4	1·0	2·4	1·1
	% Adherence	64·3		61·5		38·5		46·2		53·8		50·0	
Red meat (< 2 serves/week)													
Pro-Ex	Serves/week	1·9	1·6	1·4	1·3	1·6	1·6	1·2	1·0	1·2	1·0	1·3	1·2
	% Adherence	71·4		78·6		83·3		91·7		91·7		86·0	
Control	Serves/week	2·0	1·8	1·7	1·4	1·7	1·1	1·3	1·2	1·8	1·3	1·6	1·3
	% Adherence	71·4		69·2		76·9		84·6		69·2		75·0	
Processed meat (≤ 1 serves/week)													
Pro-Ex	Serves/week	2·0	1·8	0·4	0·7	0·8	0·8	1·4	1·4	0·8	1·1	0·8	1·1
	% Adherence	35·7		78·6		66·7		50·0		66·7		66·0	
Control	Serves/week	1·4	1·9	1·0	1·1	0·8	1·1	1·1	1·3	1·0	1·1	1·0	1·1
	% Adherence	42·9		46·2		53·8		46·2		46·2		48·1	

Pro-Ex, protein plus exercise group. All values are expressed as mean and sd or proportions (%). Adherence was calculated as the percentage of participants meeting ≥ 80 % of recommendations for each protein source as indicated. Number of participants with protein data: Pro-Ex – baseline and week 2 (*n* 14) and weeks 6, 9 and 12 (*n* 12); Controls – baseline (*n* 14) and weeks 2, 6, 9 and 12 (*n* 13). Mean represents average from weeks 2 to 12.

### Dietary intake

As shown in Table 4, both the Pro-Ex and control group had similar reductions in total energy, carbohydrate and total fat intake from baseline to 12-weeks. However, the mean daily protein intake in the Pro-Ex group increased by 13·1 g (95 % CI: −3·8, 29·9 g) after 12-weeks but decreased by 22·1 g (95 % CI: −41·4, −2·7 g) in controls, which represented a large effect (d = 1·23) for the between-group difference. While both groups reduced their total and saturated fat intake over time, this tended to be greater in Pro-Ex compared with controls (total fat: small effect d = –0·31; saturated fat: moderate effect d = –0·57).

**Table 4.** Baseline and within-group changes after 6 and 12 weeks in total energy and macronutrient intake, physical activity, 30 s sit-to-stand performance and body composition measures in Pro-Ex and controls and between-group differences for changes with effect size (Cohen’s d)

	Pro-Ex		Control		Between-group difference		Effect size
Dietary intake	Mean	sd or 95 % CI	Mean	sd or 95 % CI	Mean	sd or 95 % CI	Cohen’s d
Total energy, kJ/d							
Baseline	8104	1988	7788	1422			
Δ 6 weeks	–1388	–2504, −272	–945	–2081, 192	–443	–1952, 1065	–0·24
Δ 12 weeks	–1351	–2324, −377	–1378	–2546, −210	28	–1405, 1460	0·02
Protein, g/d							
Baseline	80·9	23·7	91·4	30·2			
Δ 6 weeks	7·0	–11·9, 25·9	–12·3	–34·2, 9·6	19·3	–8·3, 46·9	0·58
Δ 12 weeks	13·1	–3·8, 29·9	–22·1	–41·4, −2·7	35·2	11·0, 59·3	1·23
Carbohydrate, g/d							
Baseline	187·6	68·7	184·0	39·4			
Δ 6 weeks	–50·1	–90·3, −9·9	–22·9	–56·1, 10·3	–27·2	–76·1, 21·8	–0·46
Δ 12 weeks	–40·3	–85·8, 5·1	–25·6	–68·6, 17·3	–14·7	–73·7, 44·2	–0·21
Total fat, g/d							
Baseline	89·4	26·7	79·6	21·8			
Δ 6 weeks	–18·4	–40·0, 3·2	–11·1	–31·4, 9·2	–7·2	–35·2, 20·8	–0·21
Δ 12 weeks	–24·5	–44·2, −4·9	–16·0	–30·8, −1·2	–8·5	–31·8, 14·7	–0·31
Saturated fat, g/d							
Baseline	33·0	14·1	31·5	14·2			
Δ 6 weeks	–14·3	–25·0, −3·6	–6·2	–16·7, 4·2	–8·0	–22·1, 6·1	–0·47
Δ 12 weeks	–17·4	–25·5, −9·2	–9·1	–19·3, 1·0	–8·2	–20·5, 4·0	–0·57
Dietary fibre, g/d							
Baseline	27·0	11·0	20·7	8·2			
Δ 6 weeks	4·5	–7·5, 16·5	1·7	–2·1, 5·4	2·9	–8·6, 14·3	0·21
Δ 12 weeks	5·7	–4·9, 16·4	3·4	–1·4, 8·3	2·3	–8·7, 13·3	0·18
Walking min/week							
Baseline	118	132	263	232			
Δ 6 weeks	70	–23, 164	–7	153, 138	78	–93, 249	0·39
Δ 12 weeks	70	12, 127	25	–121, 172	44	–110, 198	0·24
MVPA, min/week							
Baseline	61	141	46	59			
Δ 6 weeks	49	–10, 107	43	–25, 111	6	–80, 91	0·05
Δ 12 weeks	86	–117, 290	40	–38, 118	46	–153, 245	0·19
30 s sit-to-stand, number							
Baseline	10·5	1·5	11·1	1·4			
Δ 6 weeks	1·8	0·4, 3·1	0·8	0·2, 1·4	1·0	–0·3, 2·3	0·61
Δ 12 weeks	4·0	2·4, 5·6	1·3	0·4, 2·3	2·7	0·9, 4·4	1·29
Weight kg							
Baseline	92·5	24·5	78·8	17·9			
Δ 6 weeks	–1·2	–2·0, −0·3	–0·6	–1·4, 0·2	–0·6	–1·7, 0·5	–0·43
Δ 12 weeks	–2·5	–4·1, −0·8	–0·8	–1·8, 0·3	–1·7	–3·5, 0·2	–0·76
Waist circumference, cm							
Baseline	110·4	16·2	102·1	14·1			
Δ 6 weeks	–2·3	–6·4, 1·9	–1·2	–3·6, 1·2	–1·1	–5·6, 3·4	–0·20
Δ 12 weeks	–2·8	–7·9, 2·4	–3·2	–6·8, 0·5	0·4	–5·5, 6·3	0·06

Pro-Ex, protein plus exercise group; MVPA, moderate-vigorous physical activity. All baseline values are unadjusted means and sds, while within-group changes from baseline at 6 and 12 weeks and between-group differences are reported as mean (95 % CI). Effect sizes (Cohen’s d) represent the net difference for the change between Pro-Ex minus the change for controls, divided by the baseline pooled sd. All change values are unadjusted means (95 % CI) and expressed as absolute changes from baseline.

### Physical activity

Total weekly walking time in the Pro-Ex group increased on average by ∼70 min throughout the study compared with an average −7 (week 6) to +25-minute (week 12) change in controls, which represented a small effect (d = 0·24) for the between-group difference at week 12 (Table 4). Weekly moderate-to-vigorous activity increased on average by ∼45 min in both group at 6 weeks and 86 min (95 % CI: −117, 290) in Pro-Ex and 40 min (95 % CI: −38, 118) in controls at week 12, with no sizeable effect for between-group differences (d = 0·05–0·19).

### Anthropometry and physical function

After 12 weeks, there was a mean 2·5 kg (95 % CI: −4·1, −0·8 kg) decrease in self-reported weight within Pro-Ex compared with a mean −0·8 kg (95 % CI: −1·8, 0·3 kg) change in controls, which represented a moderate effect (d = –0·76) for the between-group difference (Table 4). Both groups experienced similar mean reductions in self-reported waist circumference (Pro-Ex −2·8 cm (95 % CI: −7·9, 2·4 cm) *v*. control −3·2 cm (95 % CI: −6·8, 0·5 cm), d = 0·06) after 12 weeks. For physical function, Pro-Ex demonstrated a mean 4·0 (95 % CI: 2·4, 5·6) increase in 30-second STS number at 12 weeks compared with a mean change of 1·3 (95 % CI: 0·4, 2·3) in controls, which represented a large (d = 1·29) effect.

## Discussion

The key findings from this feasibility intervention trial in adults with NAFLD were that remote delivery and monitoring of a 12-week, home-based lifestyle program via telehealth incorporating a thrice weekly muscle strengthening program with dietary advice to increase plant-based protein intake was safe as demonstrated by the single minor adverse event (exercise related) and with high retention (89 %), but adherence in terms of meeting our predefined criteria of ≥ 66 % (exercise program) and ≥ 80 % (protein recommendations) was modest. Mean adherence to the 12-week muscle strengthening program was 52 %, and while mean daily serves of plant-based protein increased (0·9 to 1·4 per day) and animal protein decreased (1·5 to 1·2 per day) after 12 weeks, the proportion of participants meeting our recommendations for plant protein (2 serves/d) was 32 % and animal protein (1–1 ½ serves/d) was 42 %. For secondary exploratory outcomes, there was evidence for greater weight loss (moderate ES), improvements in 30 s STS performance (large ES) and changes in macronutrient intake (increased total protein intake, large ES; decrease saturated fat, moderate ES) in Pro-Ex *v*. controls after 12 weeks.

The finding that our home-based exercise program which was delivered and monitored remotely by EPs via telehealth using a smart-device exercise app was safe, is consistent with prior telehealth-based exercise studies conducted in older adults and those with chronic diseases^(37)^ including people with NAFLD^(12,38)^. However, mean adherence to the thrice weekly muscle strengthening program in our study was modest (mean 52 %) when considering all fourteen participants initially randomised to the Pro-Ex group. It is difficult to explain these findings given participants were prescribed an individually tailored exercise program and received regular monitoring and support throughout the intervention via the TeleHab app and video conferencing with the study EP, both of which have been reported to enhance adherence in previous telehealth exercise interventions in older adults including those with NAFLD^(12,39)^. Furthermore, participants received weekly behavioural support messages and exercise reminders via text and were followed up via phone and/or email by the EP if two or more consecutive sessions were missed. It is possible that the modest adherence may relate to the fact that our participants were an older (mean age 63 years) high-risk cohort in which 93 % were overweight/obese and 43 % reported having further comorbidities (e.g. type 2 diabetes, CVD). Indeed, there is evidence that adherence to home-based exercise programs in people with chronic diseases is lower compared with those with fewer health conditions and who are non-obese^(40)^. However, other telehealth-based exercise interventions conducted over 8–36 weeks in those with chronic diseases including NAFLD have reported significant heterogeneity in terms of adherence (range 1–84 %)^(12,38,41)^. This has been attributed to factors including the level of participant monitoring/ supervision and feedback provided, the mode of delivery (i.e. online web portal) not being adequately motivating for at home exercising and the length of intervention. It is worth highlighting that when we considered only participants who completed the 12-week exercise program (*n* 9), mean adherence to the muscle strengthening program was 73 %. This provides some evidence to support the feasibility of our home-based, exercise program delivered and monitored via telehealth for participants that were able to complete the study.

Another key outcome from our study was that overall adherence to the specific plant, animal and total protein recommendations was relatively modest with 14–42 % meeting our recommended targets. While this might be due in part to our relatively stringent (and high) predefined protein target levels (e.g. at least 80 % adherence), there are several other likely factors that may explain these findings. Although all participants did receive a one-on-one consultation with the study nutritionist upon study commencement, and mean attendance to group nutrition counselling sessions was 79 %, and participants were regularly monitored via protein checklists and food records, all elements which have been recognised to improve adherence during dietary interventions^(42,43)^, it would seem that this high risk cohort may have required more frequent and individualised support, especially during the second half of the intervention when adherence plateaued. In part support of this notion, prior interventions assessing Mediterranean style diets over 12-weeks, including in those with NAFLD, have reported significant dietary change with more frequent (weekly or fortnightly) dietary counselling sessions and/or regular contact via phone with the study nutritionist^(44,45)^. Despite the modest adherence to the daily/weekly protein serving targets in our study, we did observe some improvement in mean serves of plant and total plant protein, which is further reflected by Pro-Ex experiencing a net 35 g increase in protein intake and 8·2 g reduction in saturated fat relative to controls. While these findings provide some evidence that our dietary approach did elicit some changes in the type of protein consumed, it would appear participants found it difficult to transition from animal to predominately plant-based protein sources. At baseline, the mean intake of plant protein in Pro-Ex was relatively low (0·9 ± 1·1 serves/d) with only 14 % (*n* 2) of participants reporting consuming tofu/tempeh and 29 % (*n* 4) consuming > 1 serve of legumes per week, whereas the average intake of animal protein was 1·5 serves per day. There is some evidence from previous studies assessing adherence to Mediterranean style dietary patterns that dietary adherence is impacted by how different an intervention is from a participant’s usual intake^(46,47)^. This suggests that a more gradual transition may be needed when transitioning from an animal to a predominantly plant-based protein diet.

An interesting observation from our study was that although participants in Pro-Ex were not prescribed a hypocaloric diet, they did experience a mean 1·7 kg (95 % CI: −3·5, 0·2; Cohen’s d = –0·76) reduction in body weight after 12-weeks compared with controls. While these findings must be interpreted with some caution since weight was self-reported, this may relate to the finding that participants in Pro-Ex reduced their daily total energy intake by on average 1351 kJ/d, which was associated with a relative 21 % (40 g) reduction in total carbohydrate intake and a 16 % (13 g) increase in protein intake. High-protein (including plant protein) diets are known to increase satiety and the thermic effect of food^(48,49)^ and have a strong association with weight loss^(50)^. While evidence regarding the effect of different protein sources for weight loss are inconclusive^(51,52)^, a longitudinal study conducted in 1730 healthy adult men reported vegetable protein intake was inversely associated with overweight/obesity, while animal protein was positively related, suggesting protein sources may be important for weight regulation, independent of total energy intake^(53)^. Another potential factor that may have contributed to the weight loss in our study was that participants in Pro-Ex increased their weekly walking and moderate vigorous activity by 44 and 46 min, respectively, at 12 weeks relative to controls.

The finding that our 12-week intervention was also associated with an improvement in physical function (30 s STS performance) is likely due to the nature of the muscle strengthening program in which participants were prescribed exercises that targeted lower leg strength (e.g. squats, lunges). Our finding is consistent with previous research demonstrating resistance training is beneficial for improving physical function in community dwelling older adults^(54)^. More specifically, previous exercise interventions of 12 weeks have reported significant net improvements ranging from 2·5 to 3·0 relative to controls in the number of sit-to-stands completed in 30 s^(55–57)^, which is consistent with our finding (mean net 2·7 increase in the number completed in 30 s relative to controls, large effect size of Cohen’s d = 1·29). This likely represents a clinically meaningful change as there is some evidence that an improvement of 2·0–2·5 stands in an individual’s score represents a minimal detectable change to exceed measurement error^(58,59)^.

Several limitations need to be considered when interpreting the study findings. First, dietary adherence was self-reported using protein checklists which are subject to recall error. Second, anthropometric measures (e.g. weight and waist circumference) were self-measured as all assessments were completed at the participant’s home. Third, this study was not designed nor powered to detect between group differences in weight, physical activity and physical function and thus these represent exploratory findings; however, data from this study can inform sample size calculations for future trials. Finally, the two male participants (93 % were female) limit the generalisability of our findings. Future research should incorporate a stronger focus on participant support and monitoring and further individualisation of dietary recommendations, to enhance adherence to such a dietary intervention.

### Conclusion

This feasibility study indicates that in adults with NAFLD a lifestyle intervention encompassing a home-based muscle strengthening exercise program combined with recommendations for a higher protein intake from predominately plant-based sources when delivered via telehealth was safe, but adherence to the prescribed program was modest. This suggests that more intensive support from healthcare professionals may be required to elicit long-term changes related to exercise and diet for people with NAFLD. However, exploratory analysis provide some evidence that this telehealth-based approach may represent a viable model of healthcare service delivery to support weight management and improve dietary quality and physical function in adults with NAFLD. However, future large-scale, adequately powered and longer-term intervention trials including reinforcement sessions/consultations at regular intervals to enhance adherence (and a follow-up to evaluate any residual benefits) are needed to address these and related clinical outcomes to determine if it represents an effective approach for eliciting clinically meaningful improvement for this population.

## References

[1] Younossi ZM , Golabi P , Paik JM , et al. (2022) The most recent and in-depth meta-analytic assessment of the global epidemiology of nonalcoholic fatty liver disease (NAFLD). S Asia 4, 2–16.

[2] Katsiki N , Perez-Martinez P , Anagnostis P , et al. (2018) Is non-alcoholic fatty liver disease indeed the hepatic manifestation of metabolic syndrome? Curr Vasc Pharmacol 16, 219–227. 28669328 10.2174/1570161115666170621075619

[3] Huang TD , Behary J & Zekry A (2019) Non-alcoholic fatty liver disease (NAFLD): a review of epidemiology, risk factors, diagnosis and management. Intern Med J 50, 1038–1047. 10.1111/imj.14709 31760676

[4] Rinella ME , Neuschwander-Tetri BA , Siddiqui MS , et al. (2023) AASLD practice guidance on the clinical assessment and management of nonalcoholic fatty liver disease. Hepatology 17, 10–1097. 10.1097/HEP.0000000000000323 PMC10735173 36727674

[5] Arredouani A (2022) Effectiveness of Lifestyle Interventions for Nonalcoholic Fatty Liver Disease Treatment. Lifestyle-Related Diseases and Metabolic Syndrome. Intech Open. https://www.intechopen.com/chapters/83010 (accessed July 2023).

[6] Kwon OY , Choi J-Y & Jang Y (2023) The effectiveness of ehealth interventions on lifestyle modification in patients with nonalcoholic fatty liver disease: systematic review and meta-analysis. J Med Internet Res 25, e37487. 36689264 10.2196/37487 PMC9903182

[7] Stine JG , Soriano C , Schreibman I , et al. (2021) Breaking down barriers to physical activity in patients with non-alcoholic fatty liver disease. Dig Dis Sci 66, 3604–3611. 33098023 10.1007/s10620-020-06673-w PMC10321307

[8] Jaglal SB , Haroun VA , Salbach GH , et al. (2013) Increasing access to chronic disease self-management programs in rural and remote communities using telehealth. Telemed E Health 19, 467–473. 10.1089/tmj.2012.0197 PMC3696947 23570277

[9] Lim SL , Johal J , Ong KW , et al. (2020) Lifestyle intervention enabled by mobile technology on weight loss in patients with nonalcoholic fatty liver disease: randomized controlled trial. JMIR Mhealth Uhealth 8, e14802. 32281943 10.2196/14802 PMC7186867

[10] Tincopa MA , Lyden A , Wong J , et al. (2022) Impact of a pilot structured mobile technology based lifestyle intervention for patients with nonalcoholic fatty liver disease. Dig Dis Sci 67, 481–491. 33939147 10.1007/s10620-021-06922-6 PMC8090923

[11] Mazzotti A , Caletti MT , Brodosi L , et al. (2018) An internet-based approach for lifestyle changes in patients with NAFLD: two-year effects on weight loss and surrogate markers. J Hepatol 69, 1155–1163. 30290973 10.1016/j.jhep.2018.07.013

[12] Pfirrmann D , Huber Y , Schattenberg JM , et al. (2019) Web-based exercise as an effective complementary treatment for patients with nonalcoholic fatty liver disease: intervention study. J Med Internet Res 21, e11250. 30602434 10.2196/11250 PMC6746083

[13] Batsis JA , Gill LE , Masutani RK , et al. (2017) Weight loss interventions in older adults with obesity: a systematic review of randomized controlled trials since 2005. J Am Geriatr Soc 65, 257–268. 27641543 10.1111/jgs.14514 PMC5414418

[14] Cai C , Song X , Chen Y , et al. (2020) Relationship between relative skeletal muscle mass and nonalcoholic fatty liver disease: a systematic review and meta-analysis. Hepatol Int 14, 115–126. 31290072 10.1007/s12072-019-09964-1 PMC6994447

[15] Wycherley TP , Noakes M , Clifton PM , et al. (2010) A high-protein diet with resistance exercise training improves weight loss and body composition in overweight and obese patients with type 2 diabetes. Diabetes Care 33, 969–976. 20150293 10.2337/dc09-1974 PMC2858200

[16] Verreijen AM , Verlaan S , Engberink MF , et al. (2015) A high whey protein, leucine, and vitamin D-enriched supplement preserves muscle mass during intentional weight loss in obese older adults: a double-blind randomized controlled trial. Am J Clin Nutr 101, 279–286. 25646324 10.3945/ajcn.114.090290

[17] Deibert P , Lazaro A , Schaffner D , et al. (2019) Comprehensive lifestyle intervention *v.* soy protein-based meal regimen in non-alcoholic steatohepatitis. World J Gastroenterol 25, 1116–1131. 30862999 10.3748/wjg.v25.i9.1116 PMC6406181

[18] Morton RW , Murphy KT , McKellar SR , et al. (2018) A systematic review, meta-analysis and meta-regression of the effect of protein supplementation on resistance training-induced gains in muscle mass and strength in healthy adults. Br J Sports Med 52, 376–384. 28698222 10.1136/bjsports-2017-097608 PMC5867436

[19] Lim MT , Pan BJ , Toh DWK , et al. (2021) Animal protein *v.* plant protein in supporting lean mass and muscle strength: a systematic review and meta-analysis of randomized controlled trials. Nutrients 13, 661. 33670701 10.3390/nu13020661 PMC7926405

[20] Hevia-Larraín V , Gualano B , Longobardi I , et al. (2021) High-protein plant-based diet *v.* a protein-matched omnivorous diet to support resistance training adaptations: a comparison between habitual vegans and omnivores. Sports Med 51, 1317–1330. 33599941 10.1007/s40279-021-01434-9

[21] Song M , Fung TT , Hu FB , et al. (2016) Association of animal and plant protein intake with all-cause and cause-specific mortality. JAMA Intern Med 176, 1453–1463. 27479196 10.1001/jamainternmed.2016.4182 PMC5048552

[22] Alnouri F , Amar F , Atanasov AG , et al. (2021) The impact of type of dietary protein, animal *v.* vegetable, in modifying cardiometabolic risk factors: a position paper from the International Lipid Expert Panel (ILEP). Clin Nutr 40, 255–276. 32620446 10.1016/j.clnu.2020.05.017

[23] Kahleova H , Levin S & Barnard N (2017) Cardio-metabolic benefits of plant-based diets. Nutrients 9, 848. 28792455 10.3390/nu9080848 PMC5579641

[24] Alferink LJ , Kiefte-de Jong JC , Erler NS , et al. (2019) Association of dietary macronutrient composition and non-alcoholic fatty liver disease in an ageing population: the Rotterdam Study. J Gut 68, 1088–1098. 10.1136/gutjnl-2017-315940 30064987

[25] Peng H , Xie X , Pan X , et al. (2021) Association of meat consumption with NAFLD risk and liver-related biochemical indexes in older Chinese: a cross-sectional study. BMC Gastroenterol 21, 221. 34001005 10.1186/s12876-021-01688-7 PMC8127290

[26] Rietman A , Sluik D , Feskens E , et al. (2018) Associations between dietary factors and markers of NAFLD in a general Dutch adult population. Eur J Clin Nutr 72, 117–123. 28901337 10.1038/ejcn.2017.148

[27] Khazaei Y , Dehghanseresht N , Mousavi SE , et al. (2023) Association between protein intake from different animal and plant origins and the risk of non-alcoholic fatty liver disease: a case-control study. Clin Nutr Res 12, 29. 36793780 10.7762/cnr.2023.12.1.29 PMC9900076

[28] Shephard RJ (2001) ACSM’s guidelines for exercise testing and prescription Can J Appl Physiol 26, 412–413.

[29] Anania C , Perla FM , Olivero F , et al. (2018) Mediterranean diet and nonalcoholic fatty liver disease. World J Gastroenterol 24, 2083. 29785077 10.3748/wjg.v24.i19.2083 PMC5960814

[30] The National Heart Foundation of Australia (2023) Healthy Living and Eating. https://www.heartfoundation.org.au/ (accessed June 2023).

[31] Department of Health and Aged Care (2023) Physical Activity and Sedentary Behaviour Guidelines – Adults (18–64 years) – Fact Sheet. https://www.health.gov.au/resources/publications/physical-activity-and-sedentary-behaviour-guidelines-adults-18-to-64-years-fact-sheet?language=en (accessed September 2023).

[32] Craig C , Marshall A , Sjöström M , et al. (2003) International physical activity questionnaire: 12-country reliability and validity. Med Sci Sports Exerc 35, 81–95. 10.1249/01.MSS.0000078924.61453.FB 12900694

[33] Cruz-Jentoft AJ , Bahat G , Bauer J , et al. (2019) Sarcopenia: revised European consensus on definition and diagnosis. Age Aging 48, 16–31. 10.1093/ageing/afy169 PMC6322506 30312372

[34] Rees-Punia E , Rittase MH & Patel AV (2021) A method for remotely measuring physical function in large epidemiologic cohorts: feasibility and validity of a video-guided sit-to-stand test. PLoS One 16, e0260332. 34797895 10.1371/journal.pone.0260332 PMC8604329

[35] Lancaster GA , Dodd S & Williamson PR (2004) Design and analysis of pilot studies: recommendations for good practice. J Eval Clin Pract 10, 307–312. 15189396 10.1111/j..2002.384.doc.x

[36] Cohen J (2013) Statistical Power Analysis for the Behavioral Sciences. Cambridge: Academic Press.

[37] Chiang S-L , Shen C-L , Chen L-C , et al. (2020) Effectiveness of a home-based telehealth exercise training program for patients with cardiometabolic multimorbidity: a randomized controlled trial. J Cardiovasc Nurs 35, 491–501. 32511110 10.1097/JCN.0000000000000693

[38] Motz V , Faust A , Dahmus J , et al. (2021) Utilization of a directly supervised telehealth-based exercise training program in patients with nonalcoholic steatohepatitis: feasibility study. JMIR Form Res 5, e30239. 34402795 10.2196/30239 PMC8408749

[39] Daly RM , Gianoudis J , Hall T , et al. (2021) Feasibility, usability, and enjoyment of a home-based exercise program delivered via an exercise app for musculoskeletal health in community-dwelling older adults: short-term prospective pilot study. JMIR Mhealth Uhealth 9, e21094. 33439147 10.2196/21094 PMC7840282

[40] Picorelli AMA , Pereira LSM , Pereira DS , et al. (2014) Adherence to exercise programs for older people is influenced by program characteristics and personal factors: a systematic review. J Physiother 60, 151–156. 25092418 10.1016/j.jphys.2014.06.012

[41] Tabak M , Brusse-Keizer M , van der Valk P , et al. (2014) A telehealth program for self-management of COPD exacerbations and promotion of an active lifestyle: a pilot randomized controlled trial. Int J Chron Obstruct Pulmon Dis 9, 935–944. 25246781 10.2147/COPD.S60179 PMC4166347

[42] Tinker LF , Rosal MC , Young AF , et al. (2007) Predictors of dietary change and maintenance in the women’s health initiative dietary modification trial. J Am Diet Assoc 107, 1155–1165. 17604744 10.1016/j.jada.2007.04.010

[43] Carels RA , Darby LA , Rydin S , et al. (2005) The relationship between self-monitoring, outcome expectancies, difficulties with eating and exercise, and physical activity and weight loss treatment outcomes. Ann Behav Med 30, 182–190. 16336069 10.1207/s15324796abm3003_2

[44] Properzi C , O’Sullivan TA , Sherriff JL , et al. (2018) Ad libitum Mediterranean and low-fat diets both significantly reduce hepatic steatosis: a randomized controlled trial. Hepatology 68, 1741–1754. 29729189 10.1002/hep.30076

[45] Opie RS , O’Neil A , Jacka FN , et al. (2018) A modified Mediterranean dietary intervention for adults with major depression: dietary protocol and feasibility data from the SMILES trial. Nutr Neurosci 21, 487–501. 28424045 10.1080/1028415X.2017.1312841

[46] Downer MK , Gea A , Stampfer M , et al. (2016) Predictors of short- and long-term adherence with a Mediterranean-type diet intervention: the PREDIMED randomized trial. Int J Behav 13, 67. 10.1186/s12966-016-0394-6 PMC4907003 27297426

[47] Beunza JJ , Toledo E , Hu FB , et al. (2010) Adherence to the Mediterranean diet, long-term weight change, and incident overweight or obesity: the Seguimiento Universidad de Navarra (SUN) cohort. Am J Clin Nutr 92, 1484–1493. 20962161 10.3945/ajcn.2010.29764

[48] Westerterp-Plantenga MS , Lemmens SG & Westerterp KR (2012) Dietary protein–its role in satiety, energetics, weight loss and health. Br J Nutr 108, S105–S112. 23107521 10.1017/S0007114512002589

[49] Nielsen LV , Kristensen MD , Klingenberg L , et al. (2018) Protein from meat or vegetable sources in meals matched for fiber content has similar effects on subjective appetite sensations and energy intake: a randomized acute cross-over meal test study. Nutrients 10, 96. 29337861 10.3390/nu10010096 PMC5793324

[50] Hansen TT , Astrup A & Sjödin A (2021) Are dietary proteins the key to successful body weight management? A systematic review and meta-analysis of studies assessing body weight outcomes after interventions with increased dietary protein. Nutrients 13, 3193. 34579069 10.3390/nu13093193 PMC8468854

[51] Basciani S , Camajani E , Contini S , et al. (2020) Very-low-calorie ketogenic diets with whey, vegetable, or animal protein in patients with obesity: a randomized pilot study. J Clin Endocrinol 105, 2939–2949. 10.1210/clinem/dgaa336 32484877

[52] Sucher S , Markova M , Hornemann S , et al. (2017) Comparison of the effects of diets high in animal or plant protein on metabolic and cardiovascular markers in type 2 diabetes: a randomized clinical trial. Diabetes Obes Metab 19, 944–952. 28181738 10.1111/dom.12901

[53] Bujnowski D , Xun P , Daviglus ML , et al. (2011) Longitudinal association between animal and vegetable protein intake and obesity among men in the United States: the Chicago Western Electric study. J Am Diet Assoc 111, 1150–1155.e1151. 10.1016/j.jada.2011.05.002 PMC3158996 21802560

[54] Zhang Y , Zhang Y , Du S , et al. (2020) Exercise interventions for improving physical function, daily living activities and quality of life in community-dwelling frail older adults: a systematic review and meta-analysis of randomized controlled trials. Geriatr Nurs 41, 261–273. 31706592 10.1016/j.gerinurse.2019.10.006

[55] Pedersen MT , Vorup J , Nistrup A , et al. (2017) Effect of team sports and resistance training on physical function, quality of life, and motivation in older adults. Scand J Med Sci Sports 27, 852–864. 28144978 10.1111/sms.12823

[56] Amasene M , Besga A , Echeverria I , et al. (2019) Effects of leucine-enriched whey protein supplementation on physical function in post-hospitalized older adults participating in 12-weeks of resistance training program: a randomized controlled trial. Nutrients 11, 2337. 31581591 10.3390/nu11102337 PMC6835698

[57] Liao C-D , Tsauo J-Y , Huang S-W , et al. (2018) Effects of elastic band exercise on lean mass and physical capacity in older women with sarcopenic obesity: a randomized controlled trial. Sci Rep 8, 2317. 29396436 10.1038/s41598-018-20677-7 PMC5797161

[58] Gill S , Hely R , Page RS , et al. (2022) Thirty second chair stand test: test–retest reliability, agreement and minimum detectable change in people with early-stage knee osteoarthritis. Physiother Res Int 27, e1957. 35592902 10.1002/pri.1957 PMC9539496

[59] Zanini A , Crisafulli E , D’Andria M , et al. (2019) Minimum clinically important difference in 30-s sit-to-stand test after pulmonary rehabilitation in subjects with COPD. Respir Care 64, 1261–1269. 31270178 10.4187/respcare.06694

